# New Surface-Treatment Technique of Concrete Structures Using Crack Repair Stick with Healing Ingredients

**DOI:** 10.3390/ma9080654

**Published:** 2016-08-04

**Authors:** Tae-Ho Ahn, Hong-gi Kim, Jae-Suk Ryou

**Affiliations:** 1 International Sustainable Engineering Materials (ISEM) Center, Ceramic Materials Institute & Division of Advanced Materials Science Engineering, Hanyang University, 222 Wangsimni-ro, Seongdong-gu, Seoul 133-79, Korea; thahn@hanyang.ac.kr; 2 Department of Civil Engineering, Hanyang University, 222 Wangsimni-ro, Seongdong-gu, Seoul 133-79, Korea; dmkg1404@naver.com

**Keywords:** autogenous healing, surface treatment, micro-crack, repair materials, water leakage

## Abstract

This study focused on the development of a crack repair stick as a new repair method along with self-healing materials that can be used to easily repair the cracks in a concrete structure at the construction site. In developing this new repair technique, the self-healing efficiency of various cementitious materials was considered. Likewise, a crack repair stick was developed to apply to concrete structures with 0.3 mm or lower crack widths. The crack repair stick was made with different materials, such as cement, an expansive material (C_12_A_7_), a swelling material, and calcium carbonate, to endow it with a self-healing property. To verify the performance of the crack repair stick for concrete structures, two types of procedures (field experiment and field absorption test) were carried out. As a result of such procedures, it was concluded that the developed crack repair stick could be used on concrete structures to reduce repair expenses and for the improved workability, usability, and serviceability of such structures. On the other hand, to evaluate the self-healing performance of the crack repair stick, various tests were conducted, such as the relative dynamic modulus of elasticity test, the water tightness test, the water permeability test, observation via a microscope, and scanning electron microscope (SEM) analysis. From the results, it is found that water leakage can be prevented and that the durability of a concrete structure can be improved through self-healing. Also, it was verified that the cracks were perfectly closed after 28 days due to application of the crack repair stick. These results indicate the usability of the crack repair stick for concrete structures, and its self-healing efficiency.

## 1. Introduction

The development of cracks in a concrete structure is unavoidable. These cracks can be caused by several factors, such as dry shrinkage, plastic shrinkage, alkali aggregate reaction, freezing and thawing, chloride attack, poor construction work, heavy workload, and design faults, and can cause a decline in the service life of a concrete structure as well as leakage problems [1]. In addition, the cracks in concrete structures generate maintenance and repair expenses.

There are many existing concrete structure maintenance and repair methods, but they all are complicated and require much money and time [2]. The existing concrete structure maintenance and repair materials can be classified into two types: organic and inorganic materials [2,3,4]. In the case of organic materials, their elastic force is generally high due to their capacity to cover variable crack widths [4]. They have a disadvantage, though: aged deterioration occurs due to hydrolysis caused by ultraviolet-ray degradation and water leakage [3,4]. Inorganic materials, on the other hand, can improve the durability of the repaired concrete through their integration with the existing ingredients of the concrete structure [2,3,4].

Recently, many researchers have reported that new inorganic concrete structure repair materials have been developed due to the improvement of their crack conformability [5]. The concomitant repair methods, however, are very difficult to carry out and require much time and money. So, there is a need to develop a new concrete structure repair and maintenance method/material that is easier to use and is more effective than the existing ones. The self-healing of concrete may considerably reduce the concrete structure repair and maintenance costs by increasing the impermeability and/or restoring the mechanical properties of a damaged or repaired concrete structure [6]. Also, the self-healing of cracks in concrete structures can be beneficial because of the crack blocks in the crack pathway, and for the prevention of leakage. Through this self-healing phenomenon, therefore, the durability and serviceability of concrete structures can be regained [7].

The idea of self-healing is well known to many researchers worldwide. A number of reviews and studies focusing on healing agents, methods, materials, evaluating and conducting experiments on the feasibility of self-healing have been published of late [8,9,10,11,12,13,14]. Encapsulation and coating of the healing materials, use of the hollow fibers and brittle glass pipes for storing the healing material, use of the shape memory materials, and adhesive agents are well known methods of self-healing [15,16]. As such, the service life and durability of a concrete structure can be extended by using the above techniques but their effectiveness has limitations. Each technique’s performance is good in certain conditions; if those favorable conditions are changed, its performance is reduced [17]. Therefore, there is a need to develop a new technique that can be easily applied for the repair of concrete structures. In this study, the crack repair stick was developed to repair under −0.3 mm wide micro-cracks in a concrete structure at the construction site. Self-healing materials were incorporated, while making the crack repair stick in order to improve its quality. When making the crack repair stick, three different sticks (C.R.S 1–3) were manufactured considering chemical stability and healing velocity. In case of C.R.S 1, it was made with cement as a general case and C.R.S 2 was made with cement and swelling agent that may make a crack smaller because of swelling phenomenon [18] whereas C.R.S 3 was made with cement, swelling agent and an expansive material (C_12_A_7_). Table 1 shows the details of ingredients in each stick. Expansive agent is well known for self-healing materials in this field. Furthermore, it can accelerate healing velocity [18]. Therefore, this study manufactured three types of sticks in order to compare different sticks with or without healing materials. Thus, the aim here was to evaluate efficiency of the different materials used in the stick. Also, to evaluate the performance of the crack repair stick, several tests and other procedures were carried out, such as a water tightness test, observation with a microscope, relative dynamic modulus of elasticity test, water permeability test and SEM analysis. Additionally, to confirm the performance of the crack repair stick for concrete structures, field experiments were conducted.

## 2. Materials and Experimental Method

### 2.1. Materials

#### 2.1.1. Manufacture of the Crack Repair Stick

**Figure 1:**
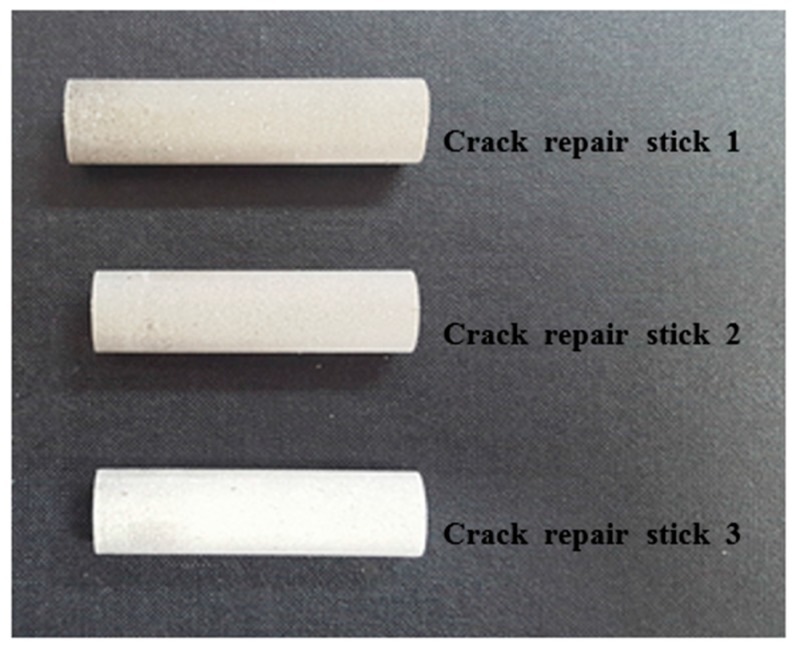
Manufactured crack repair sticks.

For repairing the cracks in a concrete structure, a crack repair stick was manufactured using inorganic materials. The crack repair stick had a 1.2 cm diameter, 5 cm length and 12~13 g weights. As mentioned above, three kinds of crack repair sticks were manufactured with various materials, and they either included or not included a healing ingredient for comparing the healing performances. The manufactured crack repair sticks were made with cementitious materials such as cement, an expansive material (C_12_A_7_), a swelling material, and calcium carbonate. For the expansive agent, the mineral-accelerator-based C_12_A_7_ from DENKA Company in Japan was used. The manufactured crack repair sticks are shown in Figure 1.

#### 2.1.2. Preparing the Specimens

**Figure 2:**
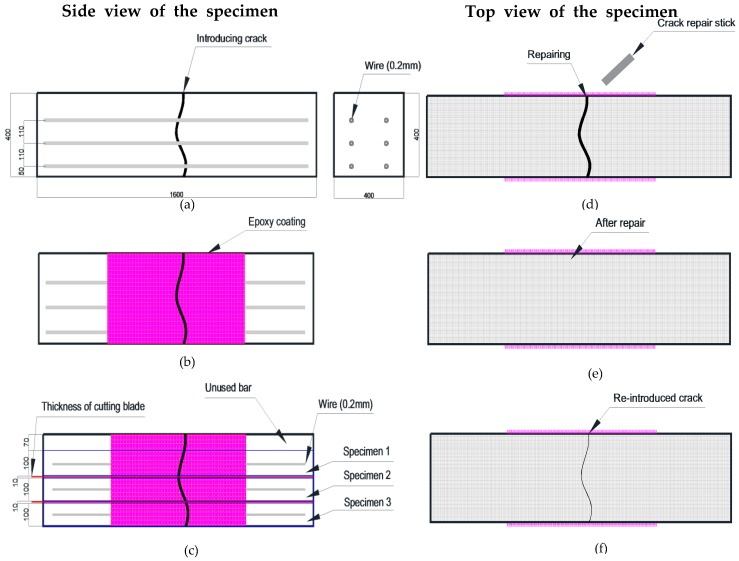
Work process of manufacturing specimens: (**a**) introducing crack; (**b**) covering epoxy at the side of the specimen; (**c**) cutting specimen in 10 mm thickness; (**d**) repairing a crack’s surface with crack repair stick; (**e**) after repairing crack; (**f**) re-introducing crack and then supplying water. (Unit: mm).

**Figure 3:**
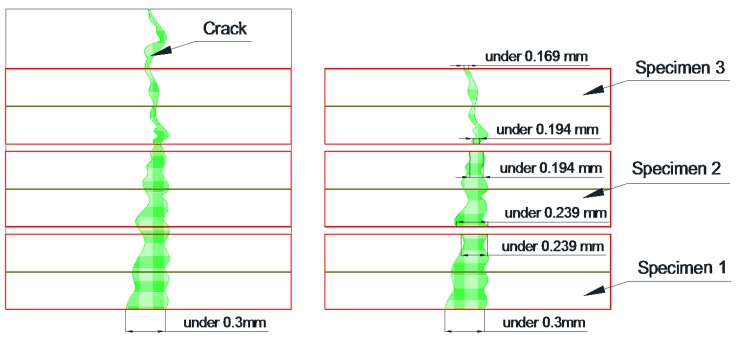
Conceptual diagrams of the crack distribution and widths of the specimen by measuring microscope.

**Figure 4:**
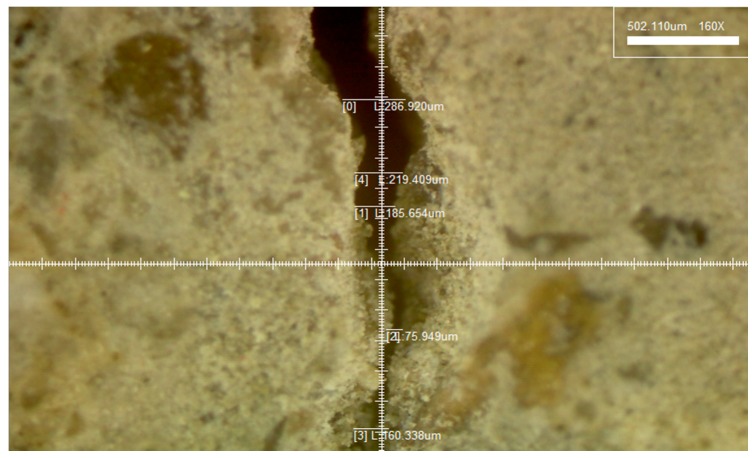
Measured microcracks by microscope.

**Figure 5:**
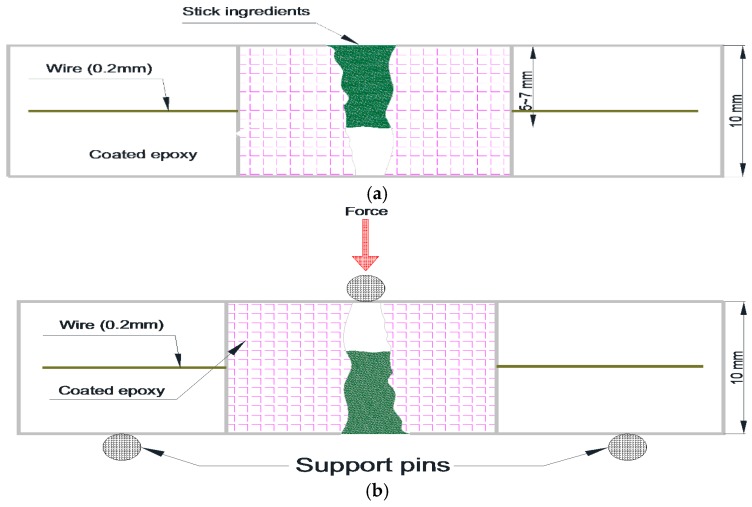

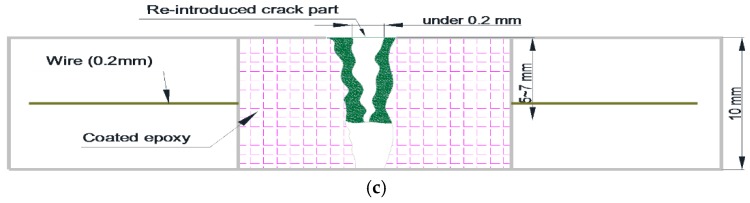
Conceptual diagrams: (**a**) depth of the stick ingredients inside crack; (**b**) re-introducing crack by three point bending machine; (**c**) crack formation after re-introducing crack.

The mortar specimens (40 × 40 × 160 mm^3^) were manufactured with 0.5 w/c ratio of and 1:3 cement-to-sand ratio according to the KS L ISO 679 standard [19]. The casting of mortars in mortar prism molds was performed in four layers of thickness: 5, 10, 10 and 15 mm. Initially, a 5 mm thick layer of the mortar was put in the mold, then two ϕ 2 mm thick wires were put longitudinally in the molds. Similarly, a 10 mm thick mortar layer was cast followed by placing two ϕ 2 mm thick wires longitudinally. Likewise, a 10 mm thick mortar layer was casted, followed by two wires and then a 15 mm thick layer. Each layer was compacted by vibration before placing the next layer. The mortar specimens were cured for 28 days in a chamber to retain a constant humidity and temperature. Under −0.3 mm wide cracks were introduced on all the specimens after curing, using a three-point bending test machine [20]. After the introduction of the cracks, the sides of the specimens were immobilized by coating them with an epoxy. Then, the specimens were cut with a diamond cutting machine (thickness of cutting blade: 1 mm) in four slices of thickness 1, 1, 1 and 0.7 cm. Each 1 cm thick slice contained two ϕ 2 mm thick wires. They were used in experiments and if slices with a thickness of 0.7 mm from the upper portion of the mortar specimen had no wire, they were discarded. Figure 2a–c shows the process of manufacturing the specimens. After that, the surface of cut specimens was repaired by using a crack repair stick. Before repairing cracks, crack widths were measured by a microscope machine. Figure 3 and Figure 4 show the crack distribution diagrams and widths measured by microscope. When measuring cracks, crack width on the top and bottom part was measured in at least five different parts and then the average value of cracks was calculated as shown in Figure 3. The measured value of micro-cracks with microscope observation is presented in Figure 5. Figure 2d–f shows the work processes that were carried out on the specimen. Thereafter, the specimens were classified into two sets for different experiments. The first set was used for the water tightness and absorption tests, and the other set was used for the dynamic modulus, water permeability and SEM tests. In the case of the self-healing specimens, the cracks on the specimens were repaired using the manufactured crack repair stick, and then the specimens were cured in a natural environment for 28 days. When repairing a crack with the stick, it was rubbed on the surface of the crack by holding it in one hand for 1–2 min approximately. When rubbing it on the surface, the stick was gently pressed against the crack. After supplying moisture, it rubs the crack surface and treats it. Figure 5 shows the conceptual diagrams of the penetration depth of the stick ingredients inside the crack after repairing the crack surface as well as on the crack formation after re-introducing the crack. As shown in Figure 5a, the ingredients of the stick went approximately 5~7 mm into the crack. Repair depth depends on the size of a crack. We confirmed this through our experiments. After the 28 days of curing, cracks were re-introduced on the previous cracks of the repaired specimens. When re-introducing cracks, a three point bending machine was used. Figure 5b shows diagrams of re-introducing crack on the specimen. After that, the widths of the re-introduced cracks were measured again by the microscope. Figure 5c shows the crack formation and width after re-introducing the cracks. Thereafter, the re-introduced cracks were supplied with water to evaluate the self-healing property and feasibility.

Also, the cracks in all the specimens that were manufactured for verifying the healing feasibility were repaired using different types of crack repair sticks to compare the possibility of using them for healing cracks. The crack was first sprayed with water to moisten it. Then, the stick was rubbed gently transversely to the length of the crack followed by rubbing on the top of crack. The speed was slow and pressure was gentle. At last, a finger was used to press any loose material inside the crack. All the procedures that were performed for this purpose are indicated in Table 2. In the case of the water tightness test, a non-repaired specimen was manufactured for comparison with the repaired specimens to confirm the blockage of water leakage before and after the repair. The self-healing property and feasibility were confirmed through various procedures.

### 2.2. Experiment Method

In this study, in order to evaluate the performance of the crack repair stick, two types of experiments were conducted; laboratory and field experiments. Lab experiments included the water tightness test, microscopic observation, relative dynamic modulus of elasticity test, water permeability test and SEM analysis. Whereas, field experiments were conducted by a field absorption test and field surface treatment test. In case of laboratory experiments, the purpose here is to evaluate the performance and efficiency of sticks after repairing the crack surface. The purpose of field experiments is to judge the effectiveness of the application of the stick to the concrete structure in the field.

#### 2.2.1. Water Tightness Test

**Figure 6:**
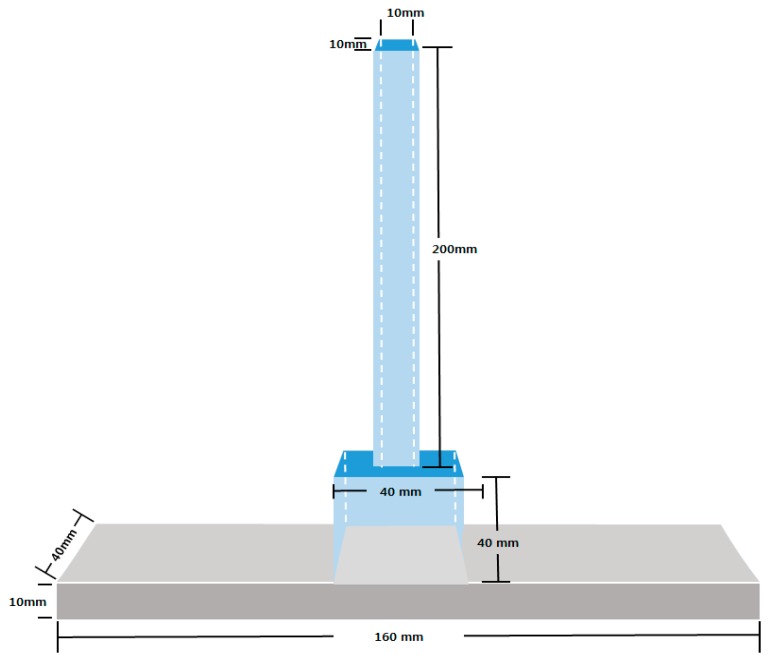
Tester of water tightness test.

In this experiment, to evaluate protection from water or other harmful ions penetration, it was conducted on the crack that had been repaired using a crack repair stick. After repairing the surface of a specimen, it was evaluated in comparison with non-repaired specimens. To evaluate the water tightness, a test set up was developed using acrylic. The tester was made of two parts: the lower part’s cross-sectional area was 40 × 40 mm^2^, the upper part’s cross-sectional area was 10 × 10 mm^2^, and their heights were 30 mm and 200 mm, respectively. Also, all the contact surfaces of the tester cubes and the specimens were sealed with epoxy to prevent leakage in the tester. Afterwards, the tester was filled with water (65 mL). The top of the cube tester was also sealed with a film to prevent water evaporation. Figure 6 shows the water tightness tester.

#### 2.2.2. Microscopic Observation

To investigate the self-healing performance and feasibility, the cracks in the specimens were observed using a microscope with 160× magnification. All the specimens that were used for the observation were prepared as a cube. Also, all the specimens were periodically supplied with water, and the re-cracks were observed after 7, 14, and 28 days, respectively.

#### 2.2.3. Relative Dynamic Modulus of Elasticity Test

**Figure 7:**
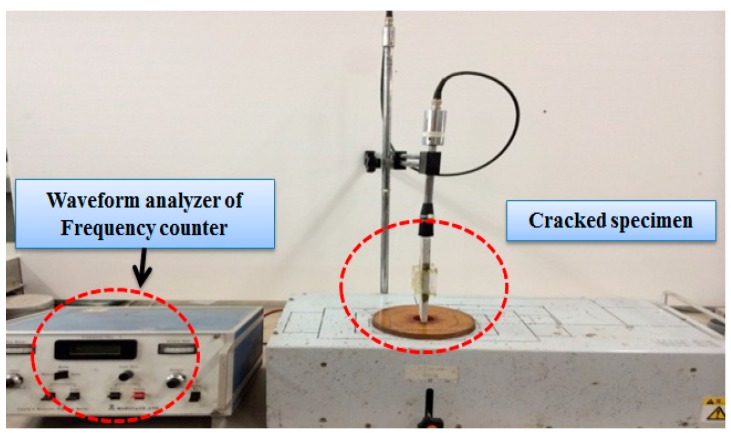
Test machine of relative dynamic modulus of elasticity.

To evaluate the durability and recovery condition of the re-introduced crack specimens, relative dynamic modulus of elasticity tests were conducted according to ASTM C215 [21]. Figure 7 shows the relative dynamic modulus of elasticity testing machine.

The equipment used for the measurement of the relative dynamic modulus of elasticity was MIN-011-0-3S from MARVI & Co., Ltd. (Seoul, Korea). The equation of relative dynamic modulus was used according to the study conducted by Lee [22].

(1) $$P_{c}=\left(\frac{{n_{c}}^{2}}{n_{0}}\right)\times 100$$

where *P_c_* represents the relative dynamic modulus of elasticity depending on the elapsed time (%), *n*_0_ represents the 1st horizontal vibration before the crack introduction, and *n_c_* represents the horizontal vibration frequency after the crack introduction depending on the elapsed time.

#### 2.2.4. Water Permeability Test

The water permeability test was conducted to verify the filling of the internal re-introduced cracks according to RILEM test Method II.4 [23]. If a specimen’s re-crack was healed, the specimen’s water permeability rate would decrease. As such, a water permeability tester was manufactured to be equal to the water tightness tester. The sizes of the manufactured water permeability testers were the same, but the tops of the testers were not sealed because it was not necessary to prevent water evaporation. To verify the differential head, the gradation at a height of 200 mm was indicated on the upper part of the tester. To calculate the water permeability, the following equation was used [22]:

(2) $$k=\frac{aL}{At}\ln\left(\frac{h_{1}}{h_{2}}\right)$$

Whereas *k* represents the water permeability coefficient (cm/s), *a* represents the cross-sectional area of the pipette (cm^2^); *L* represents the specimen thickness (cm); *A* represents the cross-sectional area of the specimen (cm^2^); *t* represents the time (s); *h*_1_ represents the initial water head (cm); and *h*_2_ represents the final water head (cm).

#### 2.2.5. SEM Analysis

**Figure 8:**
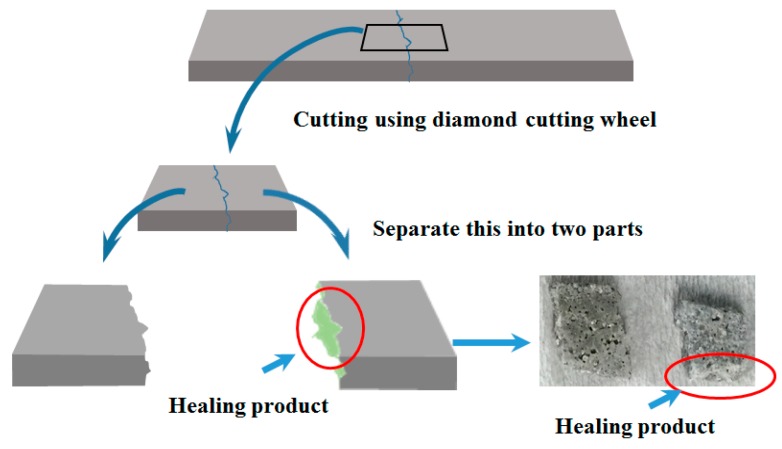
Samples for SEM.

After the cracks were repaired, the healing products that were formed inside the cracks were analyzed using SEM (Hitachi, Tokyo, Japan). In this study, a scanning electron microscope with a 0.2–30 kV accelerating voltage, a 10 × 10^−12^~10 × 10^−5^ A probe current, a 3.5 mm secondary electron imaging (SEI) resolution (WD = 8 mm; Acc = 35 kV), 10–3,000,000× magnifications, and SEI, backscattered electron image (BEI), and X-ray image modes were used to evaluate healing products. The specimens were cut at the cracked location with a diamond cutting wheel and small cubes were obtained as shown in Figure 8. SEM analysis was carried out conforming to [24]. Care was taken while taking the samples for SEM in order to avoid any damage to the products formed inside the cracks.

### 2.3. Field Experiment Method

#### 2.3.1. Field Test

**Figure 9:**
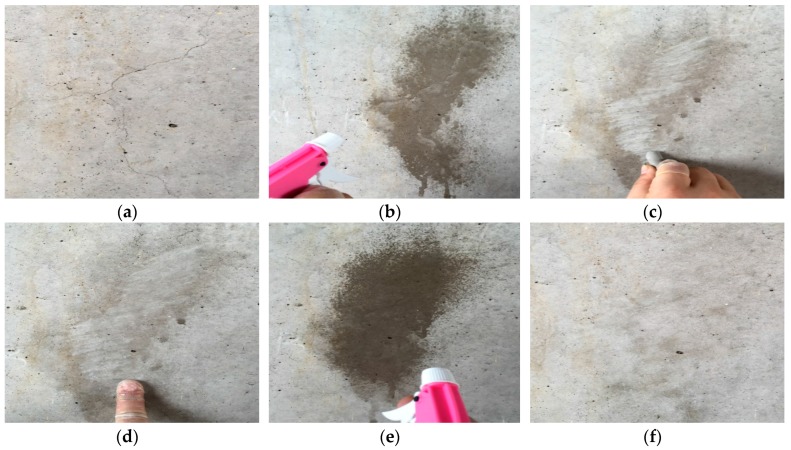
Repair procedures at the construction site. (**a**) The crack on the surface of the concrete structure at the site was selected for repair; (**b**) The crack surface was supplied by spraying water on it; (**c**) The crack surface was rubbed by using the crack repair stick first perpendicular to crack and then on its top; (**d**) The repaired surface was rubbed again by finger; (**e**) Finally, the repaired surface supplied with water again; (**f**) The repaired surface after 30 min.

To verify the performance and usability of the crack repair sticks for concrete structures, repairs were made by applying the crack repair sticks onto the specimens’ cracks. In order to evaluate the usability and efficiency of a repair stick at the site, cracks in a bridge pier near the Han River in Seoul, South Korea were repaired by using crack repair sticks. The reason for selecting this area is that the environment surrounding the river frequently subjects the repaired crack surface to moisture because of a mist. Below in Figure 9 are the steps taken:

#### 2.3.2. Field Absorption Test

In the case of the repair of the cracks in concrete structures at the site, the absorption rate of the structure must be verified to determine if penetration by harmful external elements can be prevented. In this study, to confirm the absorption rate of the concrete structure, an experimental equipment was manufactured: a tester made with two cubes, with the lower part measuring 3 × 3 cm^2^ and the upper part 1 × 1 × 30 cm^3^.

## 3. Results and Discussion

### 3.1. Water Tightness Test

**Figure 10:**
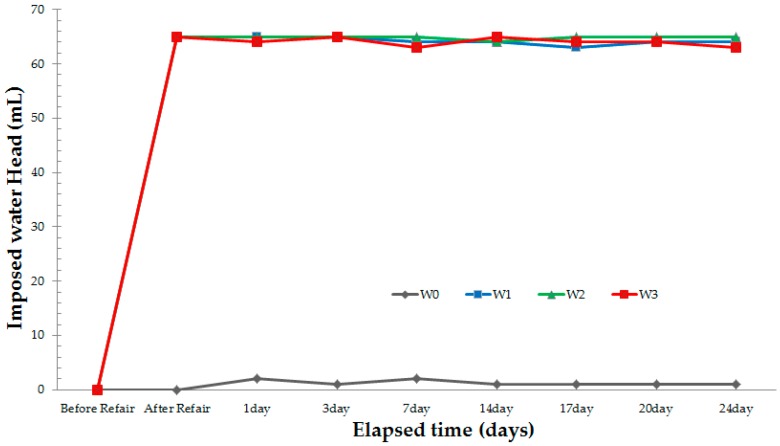
Results of water tightness test.

Two types of testers were used for the water tightness tests in this study: a non-repaired specimen and repaired specimens. Microcracks in a concrete are a potential way to decrease the durability and usability due to penetration of harmful elements in the concrete structure through them. Therefore, a water tightness test was conducted in order to confirm the possibility of penetration from outside after repairing cracks with a manufactured crack repair stick. This experiment was conducted before using the stick on the concrete structure at the site. The purpose of making cracks repair sticks is to easily repair cracks on site. However, in order to use sticks at the site, it would be necessary to confirm its effectiveness. Therefore, to evaluate the water tightness of the specimens, all the testers were filled with water (65 mL). Thereafter, the water leakage quantities before the repair and 24 days after the repair were confirmed. For the results, in the case of the non-repaired specimen, complete water leakage was observed within 1–2 min. In the case of the repaired specimens, however, no water leakage was observed. Figure 10 shows the results of the water tightness tests. The water tightness of all the repaired specimens did not change with the passage of time, but the non-repaired specimen leaked every day as soon as the tester was filled with water. These results indicate that water leakage can be prevented and that a concrete structure can be protected from penetration of harmful external ions by repairing cracks with the manufactured crack repair stick.

### 3.2. Observation Using a Microscope

**Figure 11:**
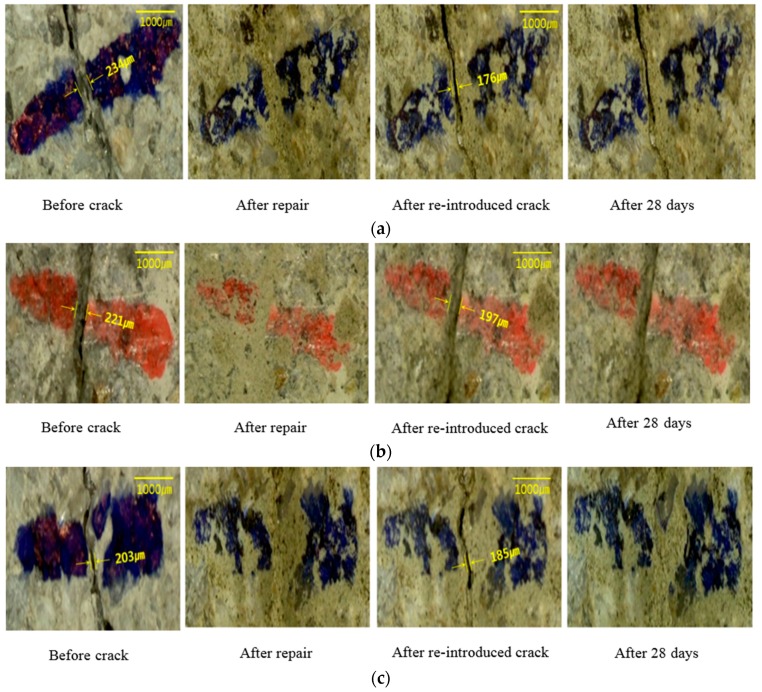
Observation of crack closure due to healing performance using microscope: (**a**) specimen by repairing C.R.S 1 and (**b**) specimen by repairing C.R.S 2 and (**c**) specimen by repairing C.R.S 3.

To observe the healing phenomenon, cracks between 0.203 and 0.234 mm were introduced in all the specimens. The cracks were repaired using the manufactured crack repair stick, and then cured in a natural environment for 28 days. After the 28 days’ curing, cracks between 0.176 and 0.197 mm were re-introduced to the repaired cracks. All the specimens to which a crack had been re-introduced were moisturized, and the parts of the surfaces with re-introduced cracks were observed for 28 days. Figure 11 shows the results of the observation with a microscope of the different specimen types: S1 (repaired with crack repair stick 1), S2 (repaired with crack repair stick 2), and S3 (repaired with crack repair stick 3). The cracks in all the specimens were filled with the crack repair stick materials after repair, and these materials were left for 28 days. The surfaces of the re-cracks of all the specimens presented different conditions compared to the initial crack surfaces. These results indicate that the crack repair stick materials remained on the surfaces of the re-introduced cracks. Based on this, it can be said that the crack repair stick can fill the inside of cracks up to approximately 5 mm wide. After the re-introduction of cracks, the re-introduced crack surfaces were observed with a microscope for 28 days. It was found that S1 and S2 had good-as-new re-crack surfaces 28 days after the repair while S3 had a changed re-crack surface 14 days after the repair. The cracks were closed, however, after 28 days. This phenomenon may be attributed to swelling and to the mixture of an expansive agent and calcium carbonate with the water in the crack, which is expected to form ettringite [18].

### 3.3. Relative Dynamic Modulus of Elasticity

**Figure 12:**
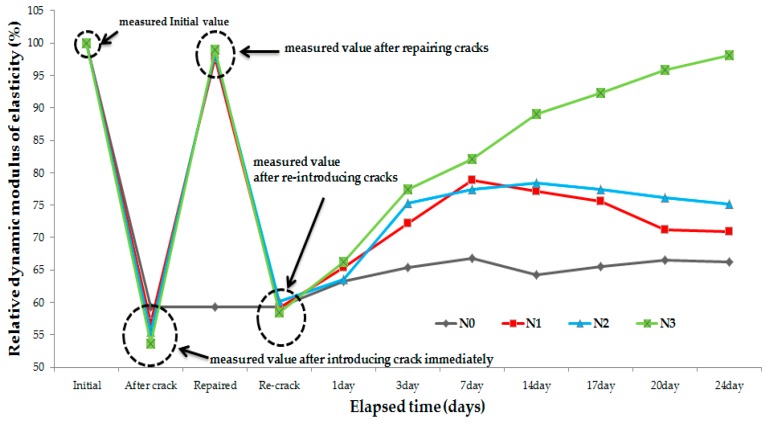
Results of the relative dynamic modulus of elasticity.

To evaluate the durability of crack filling through healing, the relative dynamic modulus of elasticity was measured before and after healing. For this, the same specimens that were used in the microscope test were used. All the specimens were cured under wet conditions for 24 days. Before measuring the relative dynamic modulus of elasticity, all the specimens were dried in a natural environment for 12 h. For the measurement procedure, first, the initial condition of the specimen was checked for comparison with the condition after the crack repair. The crack was then repaired using the crack repair stick, and then the relative dynamic modulus of elasticity was measured. Re-introduced cracks were then introduced for comparison with the conditions 1, 3, 7, 14, 17, 20, and 24 days after the repair. Figure 12 shows the relative dynamic modulus of elasticity results. These results show that the relative dynamic modulus of elasticity of all the specimens decreased after the introduction of cracks but increased by nearly 93%–95% after the repair using the crack repair stick. Based on this, it can be said that repairing cracks in concrete structures with the crack repair stick can improve the durability and serviceability of such structures. After the introduction of re-introduced cracks in the repaired specimens, however, the relative dynamic modulus of elasticity of all the specimens decreased by approximately 58%–60%. Thereafter, the cracks in all the specimens were supplied with water, and then the relative dynamic modulus of elasticity was measured after 1, 3, 7, 14, 17, 20, and 24 days. The experimental results show that the changes in relative dynamic modulus of elasticity of N0 (non-repaired specimen) were minimal during testing period. However, the relative dynamic modulus of elasticity of N1 (repaired with crack repair stick 1) increased after 7 days but did not change henceforth. In the case of N2 (repaired with crack repair stick 2), its relative dynamic modulus of elasticity increased and became greater than that of N1 after 7 days, but it did not change after 14 days. As for N3 (repaired with crack repair stick 3), its relative dynamic modulus of elasticity increased by nearly 85% after 7 days, and then increased by nearly 98% after 28 days. These results indicate that the crack repair stick containing healing agents that was used to repair the initial cracks can also be used to fill the re-introduced cracks through self-healing under wet conditions. This means that the durability of the concrete structure may be improved due to the self-healing behavior.

### 3.4. Water Permeability Test

**Figure 13:**
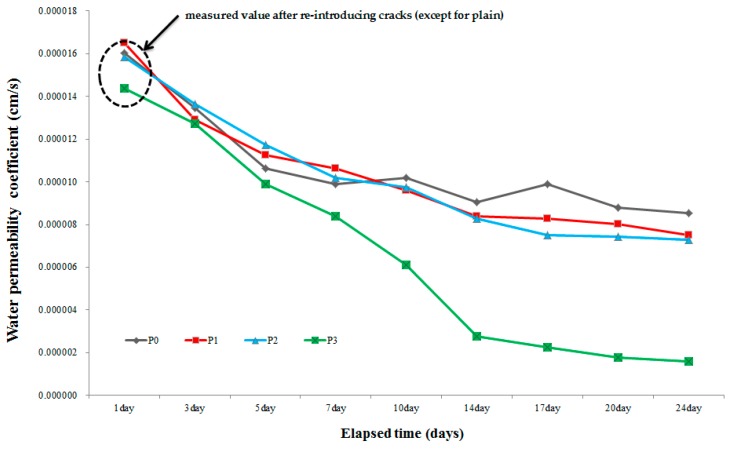
Results of the water permeability test.

To verify the degree of filling of cracks, a water permeability test was conducted in this study. Water leakage is an important degradation factor in concrete structures. If cracks occur on a concrete structure, its durability and serviceability will decrease due to water leakage [25]. Therefore, the possibility of filling a crack was evaluated. All the specimens that were used in this test were the same as those that were used for the observation with a microscope. Also, water permeability testers were used, as mentioned in Section 2.2.4. Recovery through healing prevents water movement through the crack. So, to investigate the healing in mortar, a water permeability test was conducted to investigate the recovery of cracks. Figure 13 shows the results of the water permeability test that was conducted for each specimen. The water permeability values of all the specimens after the introduction of re-cracks were 1.4 × 10^−5^–1.6 × 10^−5^ cm/s. From the results, the initial value of *P*_0_ (non-repaired specimen) was 1.6 × 10^−5^ cm/s and then it decreased to 9.8 × 10^−6^ cm/s and 8.5 × 10^−6^ cm/s after 14 and 24 days, respectively. In the case of *P*_1_ (repaired with crack repair stick 1), the initial value was 1.6 × 10^−5^ cm/s, and then it was decreased by 8.4 × 10^−6^ cm/s after 14 days but nearly did not change after 14 days. Also, the initial value of *P*_2_ (repaired with crack repair stick 2) was 1.6 × 10^−5^ cm/s, and then it was decreased by 8.3 × 10^−6^ cm/s after 14 days but also did not change after 14 days. As for *P*_3_ (repaired with crack repair stick 3), the initial value was 1.4 × 10^−5^ cm/s, and then it decreased by 2.8 × 10^−6^ cm/s after 14 days, and then it was finally decreased by 1.6 × 10^−6^ cm/s after 24 days. These results can be attributed to the self-healing ingredients of the crack repair stick. Therefore, *P*_3_ was more reactive than the other specimens because of the self-healing ingredients of the crack repair stick that was used to repair its crack (crack repair stick 3). As a result, the possibility of crack closing was verified, and it was confirmed that water leakage could be prevented and that the repair and maintenance costs could be decreased by repairing cracks simply with the use of the crack repair stick.

### 3.5. SEM Analysis

The phenomenon of self-healing has been reported by many researchers. Based on the literature [26], self-healing of cracks in cementitious materials can be divided into three different categories which are natural healing, autonomic healing and activated repairing. First, natural healing is a phenomenon in which cracks in concrete are naturally clogged in an environment with moisture. Autonomic healing is a phenomenon in which cracks are clogged with special material, such as by adding supplementary cementitious materials or accelerating the clogging of cracks in an environment involving moisture. Last, activated repairing is a phenomenon in which cracks are clogged by the mechanisms of special devices embedded in concrete for the purpose of automatically repairing cracks [26]. Jacobsen and Sellevold [27] noticed newly formed C–S–H-related portlandite and ettrigite in cracks. Schlangen and Ter Heide [28] also detected newly formed C–S–H in cracks after the cracked samples were immersed in water for 56 days. They concluded that the phenomenon of self-healing is caused by the hydration of un-hydrated cement materials [16,17]. Edvardsen [29] reported the formation of calcium carbonate in cracks. According to him, when the CO_2_ in the air is dissolved in water, CO_3_^2−^ ions diffuse into the cracks through the crack entrances. Then, calcium carbonate (CaCO_3_) precipitates in the cracks when the concentration between Ca^2+^ and CO_3_^2−^ reaches the supersaturation stage.

In this study, a crack repair stick was manufactured using cement, an expansive agent, a swelling agent, and calcium carbonate. The mineral-accelerator-based C_12_A_7_, which includes quick-setting mineral-based aluminate, takes advantage of the latter’s outstanding hydration acceleration and stable condition for strength development [30]. The main ingredients of C_12_A_7_ are CaO and solid-solution Al_2_O_3_. It has various mineral characteristics according to the composition ratio. According to such ratio, the share of C_3_A is approximately 5%–10%, which is important for setting. The other mineral agents, such as CA, CA_2_, and C_12_A_7_, are not only the main ingredients of aluminate materials but also form ettringite with gypsum. The mineral-accelerator-based C_12_A_7_ is quick-setting and hardens when mixed with water. Pure C_12_A_7_ with cement can harden the cement matrix. It produces C_2_AH_8_ and C_4_AH*_X_* (*X* = 13 or 19) when mixed with CaO [30,31]. Below is the result:

2CaO + 12CaO·7Al_2_O_3_A + 56H_2_O → 2 (2CaO·Al_2_OA·8H_2_O) *X* = 19: 16CaO + 12CaO·7Al_2_O_3_ + 133H_2_O → 7 (4CaO·Al_2_O_3_·19H_2_O) *X* = 13: 9CaO + 12CaO·7Al_2_O_3_ + 3CaSO_4_ + 224H_2_O → 7 (3CaO·Al_2_O_3_·3CaSO_4_·32H_2_O)

**Figure 14:**
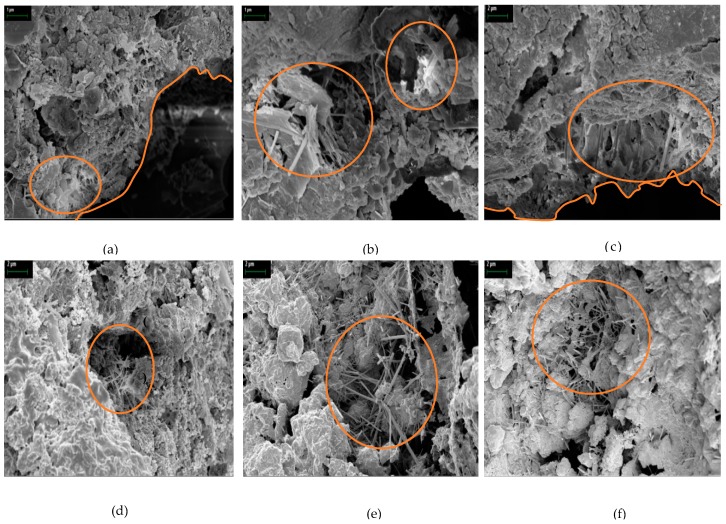
SEM micrographs of healing products on the crack surface (repaired by C.R.S 3), the scale is shown on top right corner of each figure, scale is 1 μm for (**a**,**b**) while 2 μm for (**c**–**f**).

Also, the mineral-accelerator-based C_12_A_7_ that reacted with Ca(OH)_2_ and CaSO_4_ in the cement formed an acicular C_3_A·3CaSO_4_·32H_2_ (ettringite) with high initial strength. The SEM images of the healing products around the existing surface and fractured sample are shown in Figure 14. These SEM images from different specimens (collected from specimen repaired with C.R.S 3) show that the products produced through the rehydration of the crack surfaces were primarily composed of the C–S–H, ettringite, and C–A–H phases [18,32]. Especially, according to the SEM results, there were products with ettringite around the crack surface. In such products, the main ingredients of the crack repair stick were an expansive agent and cement materials. Therefore, in the case where cement was mixed with accelerator-based C_12_A_7_, much ettringite was formed due to the existence of Ca(OH)_2_ and CaSO_4_ generated by CaO + H_2_O [18,30]. Also, the C–A–H phases were shown. Healing products in the early stage may include high Al content. Therefore, ettringite and C–A–H phases around cracks can lead to precipitation due to C_3_A and C_4_AF hydration from SEM analysis [32,33]. Also, C–S–H gel was shown that to be entangled with ettringite. C–S–H gel was formed due to the hydration of the cement particles and from the effects of Al(OH)_3_ and NaOH [22]. Consequently, various substances developed into a C–S–H gel state. Therefore, the SEM results indicate that the cracks were closed by the healing products, and that cracks repaired with the crack repair stick can be closed by these healing ingredients of the crack repair stick.

### 3.6. Field Experiment

**Figure 15:**
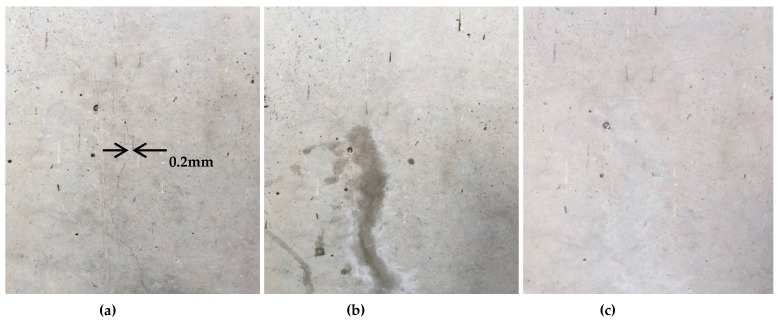
(**a**) Crack before repair; (**b**) crack after repair; and (**c**) crack after 60 days of repair.

One of the main purposes of the manufacture of the crack repair stick was for the easier and faster repair of the cracks in concrete structures at the site. Therefore, in this study, field experiments were conducted. The purpose of such experiments was to verify the usability and performance of the crack repair stick for repairing cracks in concrete structures at the site. After the repair of the cracks, the parts of the surfaces that were repaired were observed for 60 days. Figure 15 shows the results of the crack repair. These results indicate that the manufactured crack repair stick can be used more easily and quickly to repair cracks in concrete structures at the site compared to the existing repair materials and methods. Also, it was found to have several advantages, such as that it could improve concrete structures’ workability and usability and could decrease repair expenses [34,35,36]. Above all, the surfaces that were repaired were good as new, unlike the surfaces repaired using the existing repair methods. After 60 days, the surfaces that were repaired were found to have returned to their initial condition. Therefore, using the manufactured crack repair stick at the construction site can reduce concrete structures’ repair expenses and can improve the workability, usability, and serviceability of such structures.

### 3.7. Absorption Test in the Construction Field

**Figure 16:**
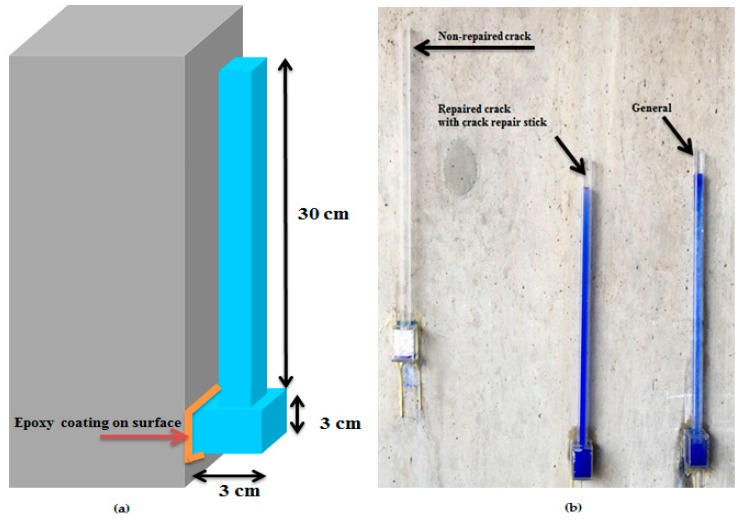
(**a**) 3D diagram of tester installed on structure; (**b**) installed testers at the site.

**Figure 17:**
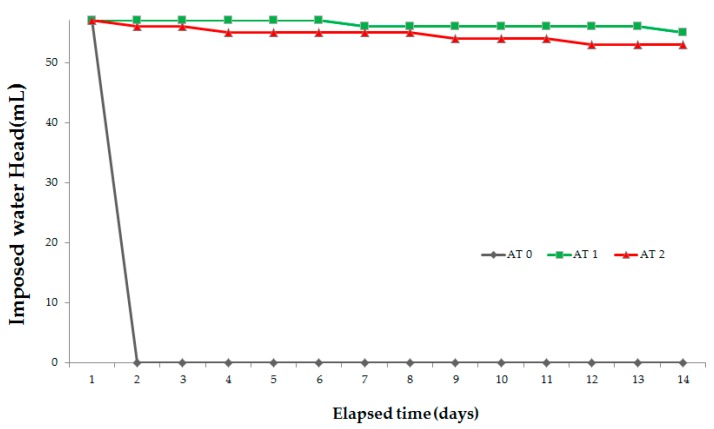
The result of absorption rates at the construction site. AT0: Non-repaired specimen, AT1: General surface, AT2: Surface repaired with crack repair stick.

To evaluate the condition of the repaired surface with the passage of time, an absorption test was conducted at a site 30 days after the repair of the concrete structure. To evaluate the repaired surface’s condition, an absorption rate tester was manufactured using acrylic. The tester was made of two parts, with the lower part measuring 3 × 3 cm^2^ and the upper part 1 × 1 × 30 cm^3^ as mentioned above. The tester was installed on the repaired surface, and then the surface was sealed with epoxy. The cube tester was then filled with a mixture of water and blue paint. To verify the absorption rate, the top of the cube tester was sealed with a coating film to prevent water evaporation. Also, to evaluate the performance of the repaired surface, two other testers were installed on the general surface with and without cracks, for comparison. Figure 16 shows the testers installed at the construction site and how to install the tester. Figure 17 shows the absorption rate results. The absorption rate values of the AT1 (general) and AT2 (repaired crack with C.R.S 3) were almost unchanged after the installation of the testers. However, the absorption rate values of the AT0 (non-repaired crack) changed by approximately 36% compared to the initial values after 15 min. Finally, the absorption rate values of the A0 decreased by 99% after 4 h. These results indicate that repairing cracks with the manufactured crack repair stick can prevent the penetration of harmful external elements. So, repairing microcracks with crack repair sticks can improve the durability of the concrete structure and can protect it from penetration by harmful external elements. Therefore, this simple and convenient repair technique can extend the life of a concrete structure.

## 4. Conclusions

In this study, a crack repair stick with self-healing ingredients was manufactured for the easy and quick repair of cracks in concrete structures at the construction site. To confirm the usability and self-healing efficiency of the crack repair stick, various experiments were conducted. Below are the conclusions that were derived based on the results:

1. The results of the water tightness tests confirmed that the water does not leak through all of the repaired specimens, but water leaked from the non-repaired specimen every day as soon as the tester was filled with water. Therefore, these crack repair sticks can block a crack and prevent water leakage as well as penetration by harmful external elements in structures.
2. Microscopic observations show that use of the above sticks repaired cracks completely. After reintroduction of cracks in repaired specimens, S3 specimens (repaired with crack repair stick 3) repaired a crack. Their crack was filled through autogenous healing of C.R.S.3 stick ingredients. While S2 experienced better recovery than S1 due to the presence of swelling agents, S1 showed low recovery as it contained only cement.
3. The relative dynamic modulus of elasticity of N1 (repaired with crack repair stick 1) increased 7 days through healing but did not change henceforth. In the case of N2 (repaired with crack repair stick 2), its relative dynamic modulus of elasticity exceeded N1 after 7 days but did not change after 14 days. In case of N3 (repaired with crack repair stick 3), its relative dynamic modulus of elasticity increased by 85% after 7 days, and then increased by nearly 98% after 28 days. Prominent recovery of the relative dynamic modulus of elasticity of N3 is due to its ingredients; expansive agent, swelling agents and calcium carbonate which were not present in N1. However, N2 showed better recovery than N1 due to utilization of swelling agent present inside it.
4. Results of the water permeability test show that *P*_1_ (repaired with crack repair stick 1) recovered its permeability from 1.6 × 10^−5^ to 8.4 × 10^−6^ cm/s after 14 days but did not change after 14 days. *P*_2_ (repaired with crack repair stick 2) recovered water permeability from 1.6 × 10^−^⁵ to 8.3 × 10^−6^ cm/s within 14 days but also did not change henceforth, whereas *P*_3_’s (repaired with crack repair stick 3) water permeability improved from 1.4 × 10^−5^ cm/s to 2.8 × 10^−6^ cm/s within 14 days and then further decreased to 1.6 × 10^−6^ cm/s. This noticeable crack recovery was due to autogenous healing; it can resist the transport of harmful ions through a repaired crack if it opens later. C.R.S.3 can sufficiently recover an open crack and thus recover the transport properties of concrete.
5. It was observed from the SEM images that the products around the existing surface and fractured parts formed during autogenous healing were primarily composed of the C–S–H, ettringite, and C–A–H phases. Especially, in the case where cement was mixed with the accelerator-based C_12_A_7_, much ettringite was formed due to the existence of Ca(OH)_2_ and CaSO_4_ generated by CaO + H_2_O. Also, the C–A–H phases showed that healing product in early stages may include high Al content. Therefore, ettringite and C–A–H phases can lead to precipitation due to C_3_A and C_4_AF hydration. Also, C–S–H gel was shown to be entangled with ettringite. C–S–H gel was formed due to the hydration of the cement particles and from the effects of Al(OH)_3_ and NaOH.
6. From field experiments, it was concluded that the crack repair stick can more easily, simply, and quickly repair cracks and it can be an alternative to the existing concrete structure repair materials/methods.

## Figures and Tables

**Table 1:** Details of the crack repair sticks.

No.	Ingredients and Their Amounts
Crack Repair Stick 1	Cement 100%
Crack Repair Stick 2	Cement 70%~80%, Swelling agent 20%~30%
Crack Repair Stick 3	Cement 40%~70%, Expansive agent 10%~20%, Swelling agent 10%~20%, Carbonates 10%~20%

Fineness modulus of expansive agent: 5000~7000 cm^2^/g, swelling agent particle size and specific gravity: pass from sieve# 200 and 2.21 g/cm^3^, fineness modulus and specific gravity of cement: 3.15 g/cm^3^ and 3410~6000 cm^2^/g.

**Table 2:** Experimental details.

Experiment Methods	Specimens	Using Materials	Condition
Water tightness test	W0	-	Non-Repaired
	W1	C.R.S 1	Repaired
	W2	C.R.S 2	Repaired
	W3	C.R.S 3	Repaired
Microscope	S1	C.R.S 1	Re-crack
	S2	C.R.S 2	Re-crack
	S3	C.R.S 3	Re-crack
Relative dynamic modulus	N0	-	Initial crack
	N1	C.R.S 1	Re-crack
	N2	C.R.S 2	Re-crack
	N3	C.R.S 3	Re-crack
Water permeability test	P0	-	Initial crack
	P1	C.R.S 1	Re-crack
	P2	C.R.S 2	Re-crack
	P3	C.R.S 3	Re-crack
Field experiment	-	C.R.S 3	Under 0.3 mm cracks
Field absorption test	-	C.R.S 3	Under 0.3 mm cracks
